# APOL1, HIV, and Pregnancy Complications: Converging Risks for Kidney Disease

**DOI:** 10.34067/KID.0000001393

**Published:** 2026-07-02

**Authors:** Michael J. Ross

**Affiliations:** Division of Nephrology, Department of Medicine, Montefiore Medical Center/ Albert Einstein College of Medicine, and Department of Development and Molecular Biology, Albert Einstein College of Medicine, Bronx, New York

**Keywords:** AIDS, apolipoprotein L1 (APOL1), HIV nephropathy, intrauterine growth, nephropathy, nephrotic syndrome, podocyte, proteinuria

Variants in the apolipoprotein L1 (*APOL1*) gene, which encodes apolipoprotein L1, are associated with an increased risk of several forms of kidney disease. High-risk APOL1 genotypes, defined by the presence of two APOL1 risk alleles (G1 or G2), are associated with a 29- to 89-fold increased risk of HIV-associated nephropathy (HIVAN).^1,2^ Although most studies have implicated a deleterious gains of function in the G1 and G2 variants that promote in kidney injury, the *APOL1* G0 (reference) allele was shown to be protective against podocyte injury in a murine model of HIVAN.^3^

Recent studies have expanded the spectrum of APOL1-associated diseases to include preeclampsia. Fetal *APOL1* high-risk genotypes are associated with preeclampsia and preterm birth.^4^ The association with fetal, rather than maternal genotype suggests an important role for placental APOL1 expression. Because preeclampsia and preterm birth are associated with reduced nephron number and increased risk of CKD later in life,^5^ it is possible that APOL1 variants may increase risk of CKD, in part, through effects on the fetus *in utero*.

In this issue, Wu *et al*. used the Tg26 murine model of HIVAN and *APOL1* transgenic mice to evaluate APOL1 variant-dependent differences in kidney function, pathology, and APOL1 expression.^6^ The study also revealed an unexpected preeclampsia-like phenotype in the G1 intercrosses that became a major determinant of the offspring phenotype.

The investigators used two previously described murine models. Because *APOL1* is not present in the mouse genome, they used transgenic mice expressing human G0 or G1 on bacterial artificial chromosomes (bacterial artificial chromosome [BAC]-*APOL1* mice). This approach preserves the endogenous human *APOL1* promoter and regulatory elements and is intended to reproduce the tissue distribution and level of human APOL1 expression.

Because mice are not susceptible to HIV-1 infection, the investigators used Tg26 mice, which carry an HIV-1 proviral transgene lacking the *gag* and *pol* genes and therefore cannot produce virions. Hemizygous Tg26 mice develop a phenotype that closely resembles human HIVAN.^7^ Because this phenotype is highly dependent on genetic background, the BAC-*APOL1* mice were backcrossed to the FVB/N background used for Tg26.

The investigators intercrossed hemizygous BAC-*APOL1*-G0 or BAC-*APOL1*-G1 mice with Tg26 mice. Each cross was expected to produce wild-type, Tg26, BAC-*APOL1*, and BAC-*APOL1*/Tg26 dual-transgenic offspring in equal proportions, permitting comparison of renal phenotypes across groups.

BAC-*APOL1*-G0/Tg26 intercrosses produced the expected distribution of offspring. In contrast, G1/Tg26 intercrosses produced fewer Tg26 and G1/Tg26 dual-transgenic offspring. These pregnancies also had lower fetal-to-placental weight ratios, and G1/Tg26 offspring had lower birth weights and higher mortality during the first 3 weeks of life.

The investigators therefore measured BP before and during pregnancy. Nonpregnant BAC-*APOL1*-G0 and BAC-*APOL1*-G1 mice had normal BP, but G1 females developed hypertension during pregnancy and 25% progressed to eclampsia. G0 females did not develop this phenotype.

*APOL1*-G0 and G1 were expressed in maternal and fetal-derived placental cells, including cells involved in remodeling maternal spiral arteries. *APOL1* mRNA had a similar distribution in human placentas, and HIV RNA was detected in both maternal and fetal-derived placental cells. In contrast, little or no *APOL1* or Tg26 transgene expression was detected in the developing kidney.

In adult *APOL1* transgenic mice, *APOL1* RNA was expressed in podocytes, endothelial cells, some parietal cells, and infiltrating immune cells, with similar levels and patterns in G0 and G1 mice. *APOL1* expression was reduced in sclerotic glomeruli, as previously reported in HIVAN.^1,2^ HIV RNA levels were similar in Tg26 and dual-transgenic mice, indicating that *APOL1* expression did not alter HIV transcription.

The pregnancy and neonatal phenotypes limited the investigators’ ability to determine whether G1 worsened adult HIVAN. Tg26 mice had increased but highly variable albuminuria, without a significant difference between Tg26 and dual-transgenic survivors. These results do not exclude the possibility that G1 worsened the HIVAN phenotype because many G1/Tg26 mice did not survive to analysis as adults.

Tg26 and G0/Tg26 mice developed similarly severe albuminuria and histologic injury, demonstrating that *APOL1* G0 did not protect against the HIVAN phenotype in Tg26 mice. Across the G0 and G1 intercrosses, G1/Tg26 offspring had lower podocyte density than G0/Tg26 offspring. Wild-type offspring from G1 intercrosses also had higher albumin excretion and lower glomerular density than wild-type offspring from G0 intercrosses. These findings suggest that the adverse intrauterine environment impaired renal development across the litter, rather than acting only through direct G1 expression in the kidneys of the offspring. These results are more consistent with a gain-of-function effect of *APOL1* risk variants than with simple loss of a protective G0 function. Although it is not clear why the investigators did not find a protective effect of *APOL1* G0 against HIV-induced injury as they had in their previous study, the disparate results may be due to differences in the HIVAN model used in that study and *APOL1* in that study was expressed under control of a podocyte-specific promoter instead of the endogenous *APOL1* promoter.^3^

Women with *APOL1* high-risk genotypes and people with HIV are at an increased risk of preeclampsia and kidney disease. This study therefore provides potentially important insight into how *APOL1* risk variants and HIV may potentiate adverse maternal and fetal outcomes. Existing murine models of preeclampsia generally use surgical manipulation or genetic alterations, and no single model fully recapitulates the human syndrome.^3^ Combining two clinically relevant risk factors may provide a useful model for studying these mechanisms.

Many models of APOL1-mediated kidney disease use high, cell-specific APOL1 expression, which can cause variant-independent injury.^4^ BAC-*APOL1* mice preserve the *APOL1* locus, including its promoter and regulatory sequences. The Tg26 model similarly uses the endogenous HIV promoter. These features reduce, but do not eliminate, the concern that supraphysiologic transgene expression caused artifactual injury.

Human *APOL1* expression in a species that lacks *APOL1* may not fully reproduce its function in humans. The Tg26 model also differs from HIV infection because every cell in Tg26 mice carries the HIV transgene, although promoter activity restricts its expression. The BAC-*APOL1* mice were generated using random transgene insertion, which can introduce artifact related to the site of integration, and potential for differences in transgene copy number. Moreover, the *APOL1* haplotypes were engineered on the EMR haplotype, which can cause non-physiologic APOL1 function.^8^

Most importantly, pregnancy loss and early mortality likely biased the G1/Tg26 group available for study in adult mice and may have masked adverse effects of G1 on kidney function in Tg26 mice. In addition, all G0 offspring but only 86% of G1 offspring were born to *APOL1*-transgenic dams, which may have diluted adverse effects in the G1 cross. Further work will be needed to separate the contributions of APOL1 expression in fetal- and maternal-derived placental tissues.

There are two primary conclusions from this study. First, *APOL1* G0 did not protect Tg26 mice from albuminuria or histologic injury, which does not support the hypothesis that *APOL1* risk variants cause kidney injury through loss of protective effects of *APOL1* G0. Second, this study suggests that *APOL1* risk variants may increase CKD risk later in life through adverse pregnancy and kidney-development effects in addition to their direct effects in adult kidney cells. Reduced nephron and podocyte endowment may lead to reduced renal reserve and increased susceptibility to CKD. Future studies are needed to further elucidate the causes of adverse pregnancy outcomes and kidney disease in people with APOL1 renal risk genotypes and in people with HIV and to identify strategies to improve their clinical outcomes.

## Supplementary Material
